# National injury prevention measures in team sports should focus on knee, head, and severe upper limb injuries

**DOI:** 10.1007/s00167-018-5225-7

**Published:** 2018-11-09

**Authors:** Malin Åman, Magnus Forssblad, Karin Larsén

**Affiliations:** 1 GIH, The Swedish School of Sport and Health Sciences, Lidingövägen 1, Box 5626, 114 86 Stockholm, Sweden; 2 Stockholm Sports Trauma Research Center, Karolinska Institutet, Box 5605, 114 86 Stockholm, Sweden; 3 Department of Surgery and Perioperative Sciences, Clinical Physiology, Umeå University, 901 85 Umeå, Sweden

**Keywords:** Sport, Accidents, Prevention, Nationwide, Medical impairment, Severe injuries, Insurance, Football, Handball, Soccer, Floorball, Ice hockey

## Abstract

**Purpose:**

To examine acute injuries in licensed floorball, football, handball, and ice hockey players in all ages nationwide in Sweden, and to identify the most common and severe injuries in each body location and recommend injury prevention measures.

**Methods:**

Using national sport insurance data from years 2006–2015 was the incidence and proportion of acute injuries, and injuries leading to permanent medical impairment (PMI), calculated in the four team sports. The most common injury type and injured body part was identified, with a particular focus of the severe injuries. Comparison between sexes was made.

**Results:**

In total, there were 92,162 registered injuries in all sports together. Knee injuries were most common, and also had the highest incidence of PMI, in all ball sports and in female ice hockey players. In male ice hockey, the most common injury was a dental and face injury, and PMI injuries were mostly in the shoulder. The most severe PMI injuries were rare and most often a face/eye injury in male floorball and ice hockey, a concussion in female ice hockey, and a knee injury in female floorball, and in both sexes in football and handball.

**Conclusions:**

To achieve the greatest impact in reducing the adverse effects of acute sport injuries nationwide in Sweden, preventive measures should focus on knee injuries in all the investigated team sports. The severe head/face and upper limb injuries also need attention. Protective equipment, neuromuscular training programs, rules enforcements, and fair-play interventions may reduce the incidence of injuries.

## Introduction

Team sports, such as floorball, football (soccer), handball, and ice hockey, are sports with high numbers of players, and account for a large proportion of the acute sports injuries reported nationally in Sweden [22, 31, 32]. Acute injuries that need medical attention and treatments are of particular importance as they may, in worst-case scenarios, cause a permanent medical impairment (PMI) [23, 25, 31]. Such injuries lead to suffering, absence from sport and high costs for society. It is, therefore, suggested that the more severe the injuries sustained, the higher the priority should be to prevent these injuries, regardless of injury incidence [25].

In Sweden, four Sports Federations (SF) (floorball, football, handball, and ice hockey) had, at the time of this study, their mandatory accident insurance in the same insurance company, covering all licensed players in all ages and levels of play. This database has previously been used to evaluate the incidence and body location of acute injuries in different sports [7, 31, 32]. The most recent study indicated that head/neck, and upper and lower limb injuries were both common and also involved a risk of PMI [32]. This study also reported that there was an increase in injury incidence in all sports except for football between 2006 and 2013 [32]. To further understand the injury pattern, especially for the severe injuries, further analyses of the reported injuries in the four sports are needed. The objective of the present study was to identify the specific body part that was most often injured and involved the most severe injuries leading to PMI in licensed players in floorball, football, handball, and ice hockey. Another purpose was to propose prevention measures for these injuries. Since the present study includes injury data from all licensed players in a whole nation, it will add knowledge about where national injury prevention measures must focus to reduce the adverse effects of sports injuries in team sports nationwide.

## Materials and methods

An observational study was set up using national insurance claims data. The total numbers of licensed athletes in each sport was obtained from the Swedish Sports Confederation (RF) for each year from 2006 to 2015. All licensed players were insured by the Swedish insurance company Folksam, through their SF, and the accuracy of the injury database has earlier been evaluated [30]. The Swedish Ice Hockey Association changed insurance company 2013; hence, no ice hockey injuries were available for the analysis after 2012. After an injury has occurred, the athlete has 3 years to report the injury to the insurance company. However, in rare cases, up to 10 years is allowed [30]. Mean (± SD) time from injury date to reporting date was 2 ± 6 month (range 0–10 years) during the study period.

The degree of permanent medical impairment (PMI) was determined by the insurance company within 1–2 years after the injury occurred and graded between 1 and 99% [31]. Thus, the analysis includes injuries that occurred between 2006 and 2013. A PMI injury results in persistent symptoms affecting activities of daily living, such as loss of motion, instability, and pain. For example, PMI after an anterior cruciate ligament (ACL) injury could be graded between 1 and 17%. The measurements PMI 1 + were calculated to include all PMI injuries (1–99%), and PMI 10 + include only those PMI injuries graded at 10% or more [32].

An acute injury is defined as acute physical damage to an athlete resulting from a specific identifiable event related to a sports activity organized by sports federation or sports club [8], and is reported to the insurance company [31, 32]. Body location, specific body part, and injury type were classified according to the existing consensus reports in sport injury research, for example in football [8]. Sprains, dislocations, strains, ruptures, and tears were combined into the same category, henceforth called “sprain/rupture” (SR). Contusions, hematomas, bruises, lacerations, and abrasions were combined into another category, hereafter called “contusion/laceration” (CL) [32]. Dental injuries and concussions were classified as separate injury types [8]. Missing injury information was quality checked with the text files within the insurance database [31, 32]. Unknown injury locations were registered as between 0.05 and 0.1% of the total injury claims in each sport.

The project was approved by the Regional Ethical Committee in Stockholm (Dnr 2012/1436-31/1).

### Data analysis

Descriptive statistics with frequencies, percentages (% of total injuries in each sport and sex), and incidences of injury were performed, using SPSS statistical software (version 21.0, Inc. Chicago, IL, USA). Injury incidence was calculated for total injuries, PMI 1 + and PMI 10+, by matching the total number of licensed athletes with the total number of unique injury claims reported to the insurance company. Injury incidence was defined as “injuries per 1,000 licensed player years”, further expressed as 1000 player years. Injury risk, expressed as risk ratio (RR) between sexes, was calculated by dividing the incidence of injury in females by the incidence in males. The material includes the total population of licensed players in the nation. Measurement accuracy is related to the numbers of meaningful digits which in this manuscript is related to the number of reported injuries (1–5 digits), and the number of athletes in each sport (4–6 digits). The numbers of licenses within each sport are reported from the SFs in integer, except for 4 years reported from the handball SF (50 and 500).

## Results

There were 92,162 registered injuries in total for all four sports together (70,538 male injuries and 21,624 female injuries) during the study period (2006–2015, and in ice hockey 2006–2012). Mean (± SD) age of the injured males was 21 ± 7 and 19 ± 6 years for females. The highest proportion of injuries was in the 15–17-year-old group, but, for male ice hockey players, it was in the 18–20-year-old group.

The knee was the most frequently injured body part in the majority of the investigated players, except in male ice hockey players where dental injuries were the most common injury (Table 1). Females had a higher RR for knee injuries in all sports, especially in floorball (RR 2.2) and handball (RR 2.0) (Table 1), SR were the most common injury type in the knee, the hand/finger was most commonly fractured, while the shoulder was most commonly fractured or had a SR.

**Table 1:** Injury incidence and number of injuries

	Floorball					Football				
	Male	No	Female	No	RR	Male	No	Female	No	RR
Head/neck										
Cervical spine	0.1	91	0.2	61	*1.9*	0.1	219	0.2	146	*1.9*
Face (incl. eye)	1.2	1052	1.1	356	*0.9*	1.6	3665	0.9	669	*0.5*
Dental	1.3	1121	1.1	354	*0.9*	1.4	3075	0.9	689	*0.6*
Head/brain	0.3	232	0.5	151	*1.8*	0.6	1237	0.6	431	*1.0*
Upper limb										
Shoulder	0.5	467	0.4	133	*0.8*	1.1	2561	0.5	414	*0.5*
Upper arm	0.02	18	0.02	7	*1.1*	0.05	104	0.03	24	*0.7*
Elbow	0.1	82	0.1	44	*1.5*	0.1	322	0.2	121	*1.1*
Forearm	0.1	106	0.1	44	*1.1*	0.3	569	0.2	154	*0.8*
Wrist	0.2	165	0.3	85	*1.4*	0.6	1281	0.6	455	*1.0*
Hand/finger	0.7	622	0.6	204	*0.9*	1.7	3756	1.1	876	*0.7*
Trunk/back										
Sternum/ RIB	0.2	164	0.2	63	*1.1*	0.3	693	0.2	167	*0.7*
Abdomen	0.1	63	0.04	13	*0.6*	0.1	236	0.1	39	*0.5*
Lower back/pelvis	0.05	44	0.1	21	*1.3*	0.1	163	0.1	73	*1.3*
Spine undefined	0.04	33	0.02	8	*0.7*	0.1	140	0.04	33	*0.7*
Lower limb										
Hip/groin/thigh	0.1	131	0.1	45	*0.9*	0.4	880	0.2	141	*0.5*
Knee	2.2	1962	4.8	1550	*2.2*	5.2	11,527	7.8	5986	*1.5*
Lower leg/achilles	0.3	237	0.2	72	*0.8*	1.1	2403	0.6	492	*0.6*
Ankle	0.5	409	0.9	284	*1.9*	1.3	2852	1.2	953	*1.0*
Foot/toe	0.4	361	0.4	139	*1.1*	1.0	2237	0.7	571	*0.7*
Total injuries	8.3	7424	11.3	3657	*1.4*	17.1	38,235	16.2	12,523	*0.9*
Specific										
Eye (face)	0.4	353	0.4	120	*0.9*	0.1	311	0.1	98	*0.9*
Concussion (head/brain)	0.2	180	0.4	134	*2.1*	0.4	822	0.5	349	*1.2*

	Handball					Ice hockey				
	Male	No	Female	No	RR	Male	No	Female	No	*RR*
Head/neck										
Cervical spine	0.4	34	0.5	36	*1.3*	1.0	427	1.4	33	*1.3*
Face (incl. eye)	5.2	462	4.5	324	*0.9*	7.8	3216	1.1	27	*0.1*
Dental	5.6	493	4.2	308	*0.8*	9.3	3818	3.3	78	*0.4*
Head/brain	1.3	115	1.6	117	*1.2*	2.6	1087	3.7	88	*1.4*
Upper limb										
Shoulder	3.9	346	2.4	174	*0.6*	6.0	2469	2.6	62	*0.4*
Upper arm	0.2	18	0.2	14	*0.9*	0.3	123	0.1	< 5	*0.3*
Elbow	0.7	62	0.9	68	*1.3*	0.5	203	0.5	12	*1.0*
Forearm	0.3	27	0.4	28	*1.3*	2.0	816	0.5	13	*0.3*
Wrist	1.1	98	1.6	113	*1.4*	2.7	1125	1.6	37	*0.6*
Hand/finger	9.4	829	8.9	643	*0.9*	3.8	1575	1.7	41	*0.5*
Trunk/back										
Sternum/RIB	0.5	47	0.6	45	*1.2*	1.1	450	1.3	31	*1.2*
Abdomen	0.2	19	0.1	7	*0.4*	0.4	175	0.2	5	*0.5*
Lower back/pelvis	0.2	22	0.3	21	*1.2*	0.3	138	0.3	8	*1.0*
Spine undefined	0.2	19	0.2	16	*1.0*	0.2	80	0.4	9	*2.0*
Lower limb										
Hip/groin/thigh	0.7	58	0.5	35	*0.7*	1.0	414	0.4	9	*0.4*
Knee	10.5	924	20.7	1504	*2.0*	4.7	1951	5.3	126	*1.1*
Lower leg/achilles	1.5	131	1.0	76	*0.7*	0.9	392	0.6	14	*0.6*
Ankle	2.4	207	2.4	172	*1.0*	1.2	513	1.7	41	*1.4*
Foot/ toe	1.8	155	1.4	104	*0.8*	0.9	378	0.6	15	*0.7*
Total injuries	46.5	4093	52.9	3837	*1.1*	47.4	19,539	28.0	665	*0.6*
Specific										
Eye (face)	0.6	52	0.4	28	*0.7*	0.4	178	0.1	< 5	*0.2*
Concussion (head/brain)	1.3	85	2.0	101	*1.5*	2.3	960	3.1	73	*1.3*

The quantities given in italic refer to the risk ratio (RR) between sexes (female injury incidence divided by male injury incidence)

Total injury incidence per 1000 player years. Number of injuries (no) during the study period, 2006–2015 in floorball, football, and handball, and 2006–2012 for ice hockey

### Dental injuries

The proportion of dental injuries was generally high in all sports, and higher in males compared to females. In male hockey players, 20% of their injuries were dental injuries, while 15% of male floorball injuries and 12% of male handball injuries were dental. Male ice hockey players younger than 18 years of age had a lower proportion of dental injuries (9.5% of total injuries) compared to players older than 18 years (27.2%). This was neither seen in female players nor in other sports.

### Permanent medical impairment (PMI)

Total PMI 1 + injury incidences in each body part and sport are shown in Table 2. Approximately 80% of the PMI injuries were graded between 1 and 4%. The more severe PMI 10 + injuries were generally rare (less than 3% of all PMI injuries) with mean incidence of 0.04 PMI 10 + injuries/1000 player years in male players and 0.03 PMI 10+/ 1000 player years in females.

**Table 2:** PMI injury incidence, numbers, and proportion of total injuries

PMI 1+	Floorball					Football				
	Male	No	Female	No	*RR*	Male	No	Female	No	*RR*
Head/ neck										
Cervical spine	0	0	0.004	< 5		0.01	8	0.02	9	*3.2*
Face (incl. eye)	0.04	25	0.02	< 5	*0.4*	0.1	98	0.05	26	*0.8*
Dental	0.001	< 5	0	0		0.003	< 5	0.004	< 5	*1.4*
Head/brain	0.003	< 5	0.003	6	*1.0*	0.01	21	0.01	6	*0.8*
Upper limb										
Shoulder	0.1	38	0.04	10	*0.7*	0.2	241	0.1	36	*0.4*
Upper arm	0	0	0.004	< 5		0.01	9	0	0	*0.0*
Elbow	0.01	9	0.01	< 5	*0.9*	0.03	40	0.02	10	*0.7*
Forearm	0.003	< 5	0.02	< 5	*5.6*	0.03	50	0.01	5	*0.3*
Wrist	0.01	7	0.02	< 5	*1.6*	0.1	104	0.1	31	*0.9*
Hand/finger	0.1	47	0.1	19	*1.1*	0.3	417	0.2	102	*0.7*
Trunk/back										
Sternum/RIB	0.001	< 5	0.004	< 5	*2.8*	0.003	< 5	0.01	< 5	*2.2*
Abdomen	0	0	0	0		0.01	9	0	0	
Lower back/pelvis	0	0	0	0		0.001	< 5	0.01	< 5	*11.5*
Spine undefined	0	0	0	0		0.002	< 5	0.002	< 5	*1.0*
Lower limb										
Hip/groin/thigh	0.01	< 5	0.004	< 5	*0.7*	0.02	30	0.01	8	*0.8*
Knee	0.4	316	1.2	312	*2.8*	1.3	2072	2.3	1247	*1.7*
Lower leg/Aachilles	0.02	13	0.03	8	*1.7*	0.2	280	0.1	51	*0.5*
Ankle	0.02	18	0.03	22	*1.7*	0.1	167	0.1	65	*1.1*
Foot/toe	0.01	8	0.03	8	*2.8*	0.1	107	0.1	31	*0.8*
Total PMI 1+	0.7	495	1.6	406	*2.3*	2.4	3675	3.1	2802	*1.3*
Specific										
Eye (Face)	0.02	16	0.004	< 5	*0.2*	0.01	23	0.01	5	*0.6*
Concussion (head/brain)	0.001	< 5	0.02	6	*16.7*	0.01	12	0.01	5	*1.2*

PMI 1+	Handball					Ice hockey				
	Male	No	Female	no	*RR*	Male	No	Female	No	*RR*
Head/neck										
Cervical spine	0.01	< 5	0.03	< 5	*2.5*	0.04	17	0.04	< 5	*1.0*
Face (incl. eye)	0.1	6	0.2	10	*2.1*	0.2	74	0.3	6	*1.4*
Dental	0	0	0.02	< 5		0.02	10	0	0	
Head/brain	0.1	5	0.02	< 5	*0.2*	0.04	16	0.1	< 5	*2.2*
Upper limb										
Shoulder	0.7	47	0.5	27	*0.7*	0.7	289	0.1	< 5	*0.2*
Upper arm	0	0	0.02	< 5		0.03	13	0	0	
Elbow	0.1	7	0.2	9	*1.6*	0.04	18	0.04	< 5	*1.0*
Forearm	0.04	< 5	0.1	< 5	*1.2*	0.1	48	0.04	< 5	*0.4*
Wrist	0.2	12	0.1	8	*0.8*	0.3	115	0.3	6	*0.9*
Hand/finger	1.4	104	1.2	69	*0.8*	0.4	177	0.1	< 5	*0.2*
Trunk/back										
Sternum/RIB	0.01	< 5	0.02	< 5	*1.2*	0.04	15	0.1	< 5	*3.5*
Abdomen	0	0	0	0		0.01	5	0	0	
Lower back/pelvis	0.01	< 5	0.02	< 5	*1.2*	0.01	5	0	0	
Spine undefined	0	0	0.02	< 5		0.002	< 5	0	0	
Lower limb										
Hip/groin/thigh	0	0	0.02	< 5		0.04	17	0	0	
Knee	2.3	168	5.7	336	*2.5*	0.6	230	0.7	17	*1.3*
Lower leg/achilles	0.1	7	0.1	8	*1.4*	0.1	39	0	0	
Ankle	0.2	13	0.2	12	*1.1*	0.1	38	0.2	< 5	*1.8*
Foot/toe	0.1	5	0.1	< 5	*1.0*	0.1	33	0.04	< 5	*0.5*
Total PMI 1+	5.3	381	8.5	498	*1.6*	2.8	1170	2.0	47	*0.7*
Specific										
Eye (face)	0.03	< 5	0	0		0.04	16	0.04	< 5	*1.1*
Concussion (head/brain)	0.04	< 5	0.02	< 5	*0.4*	0.04	15	0.04	< 5	*1.2*

The quantities given in italic refer to the risk ratio (RR) between sexes (female injury incidence divided by male injury incidence)

PMI 1+ (permanent medical impairment degreed 1–99%). Injury incidence per 1000 player years. Number of injuries (no.). Study period 2006–2013 for floorball, football, and handball, and 2006–2012 for ice hockey. Risk ratio for females compared to males

### Floorball

PMI 1 + injuries were most often an SR injury to the knee in both sexes (5% of total male injuries and 10% of total female injuries). A hand/finger fracture was the second most common PMI 1 + injury in males (0.8%) and ankle fracture for females (0.7%) (Table 2).

PMI 10 + injuries were rare, approximately 2 each year. Incidence in males was 0.04/1000 player years, and in females 0.02/1000 player years. Most often, it was an SR injury to the knee in female players (0.02/1000 player years). In male floorball players, the eye had the greatest PMI 10 + incidence (0.01/1000 player years), which was the highest incidence in all sports in this study. The most severe PMI (75–100%) were head/brain injuries (male floorball players). There was a minor decrease in knee PMI 1 + injuries in both sexes (from 0.6 to 0.4 injuries/1000 player years in males and from 1.3 to 0.8 injuries/1000 player years in females).

### Football

PMI1 + was most often an SR injury to the knee in both sexes (7% of total male injuries and 12% of total female injuries). A hand/finger fracture was the second most injured in both sexes (1% in each sex). PMI10 + injuries were rare, 0.04/1000 player years in both males and females. Most common was an SR injury to the knee in both sexes (0.02/1000 players years), followed by a face (eye) injury in males (0.005/1000 player years) and an SR injury to the ankle or cervical spine in females (0.004/1000 player years). There was a decrease in knee PMI 1 + injuries, from 1.4 to 0.8 injuries/1000 player years in males and from 2.8 to 1.0 injuries/1000 player years in females.

### Handball

The most common PMI 1 + injury in both sexes was an SR injury to the knee (5% of total male injuries and 11% of female injuries), followed by a hand/finger fracture (3% of male and 2% of female injuries). PMI 10 + injuries were very rare, less than one injury each year (0.06/1,000 player years in males and 0.03/1000 player years in females). In males, the reported PMI 10 + injuries were an SR injury to the knee, CL in the eye, concussion, or an SR injury to the shoulder (each 0.03% of total male injuries). In females, it was an SR injury to the knee or the shoulder (each 0.03% of total female injuries). There was a minor decrease in knee PMI 1 + injuries, from 3.2 to 2.1/1000 player years in males, and from 4.8 to 3.9/1000 player years in females.

### Ice hockey

PMI 1 + injuries were most often an SR injury to the shoulder in males (2% of total male injuries) and to the knee in females (3% of total female injuries). Second most commonly occurring injury was an SR injury to the knee in males (1%) and a wrist fracture in females (1%). PMI 10 + injuries were very rare in female players, less than five occurred during 8 years, giving an incidence of 0.04/1000 player years. Most common PMI 10 + injury type was concussion (0.2%). Male players exhibited the same injury incidence, but injury location was the eye or an SR injury to the shoulder or to the knee (each 0.02%). Among male hockey players, there was a registered spinal cord injury at the thoracic level (0.01%), leading to a PMI graded between 75–100%.

Female ice hockey players had the highest incidence of all concussions leading to PMI, both in total PMI 1 + and PMI 10+. However, there were less than five PMI 10 + concussion injuries during the study period.

### Injury incidence over time

Total injury incidence decreased during the study period (2006–2015) in all sports except ice hockey where there was an increase in injury incidence by 20% in males and 37% in females (2006–2012). Football players had a constant decrease in injury incidence over the study duration (22% in males and 32% in females). Floorball injury incidence decreased by 4% in males and 15% in females. Handball injuries decreased by 6% in males and 7% in females. Floorball and handball reported an increase in injury incidence until 2013, and then a decrease from 2013 to 2015.

The decrease in injury incidence in the three ball sports was mostly seen as a decrease in knee and dental injuries. Total knee injury incidence decreased to some extent in all the three ball sports between 2006 and 2015: 33% in football, 7% in handball, and 9% in floorball. The two latter sports had an increase in knee injury incidence until 2013, and then a decrease from 2013 to 2015. Dental injury incidence decreased in all players during the studied period, except in female ice hockey players who already had low incidences of these injuries.

## Discussion

The main findings of the present study were that the knee had the highest incidence of both acute injury and PMI 1 + in all ball sports, for both sexes. This has been recognized in the earlier studies [6, 9, 18, 27]. In addition, females had higher RR of knee injury incidence compared to males in the investigated sports, which has also been shown in the other studies, even higher among severe knee injuries [16, 18, 20, 28]. In general, the proportion of knee, ankle, and foot/toe injuries was higher in females compared to males in the present study.

### Floorball

Floorball is played indoor, surrounded by 50 cm high boards. The players use a smaller and much lighter stick compared to ice hockey players and a light plastic ball. The ball can reach speeds of up to 200 km per hour. Only the goal keeper wears protective equipment. In the present study, knee, face (including eye injuries), and dental injuries were the most common injuries among floorball players, with PMI 1 + occurring particularly at the knee. There are a few studies on floorball, but those that exist have found the same injury patterns as in the present study [18, 21]. Foorball players had the highest proportion of eye injuries in the sports investigated in the present study (3–5% of total injuries), and this has also been reported in the other studies [4, 21]. These eye injuries have been found, in the earlier studies, to be caused by being hit by a ball or stick [4, 21]. One recent study has highlighted the importance of protective eyewear to prevent floorball-related eye injuries [4], and in 2015, it became mandatory for youth players (younger than 15 years) to wear eye protection in Sweden. Hopefully, this rule change for youth players will reduce eye injuries in floorball, but this needs further investigation.

The decrease in knee injuries after 2013 may, to some extent, be explained by gradual introduction of a neuromuscular training (NMT) program to Swedish floorball players. A similar program has led to a reduction in leg injuries in floorball players in Finland [19], but further research is warranted.

### Football

The more severe injuries in football were SR injuries to the knee and hand/finger fractures, for both sexes. Knee injury is a well-reported severe injury in football in all the ages [5, 10, 23]. In the present study, as in others, it is found that younger players experience more fractures and upper limb injuries than older ones [6]. Fractures require medical treatment, and there for medical costs for the player. Thus, it is more likely that this type of injury is reported to the insurance company than others. During the study period, football players had a decrease in lower limb injuries, particularly in the knee, as well as in dental injuries. The effect of the Swedish NMT “Knee control program” [26], structurally implemented nationwide in Sweden from 2010, to reduce knee and CL injuries, may explain the decrease in knee injuries, but further research is needed.

### Handball

The most common injuries among handball players were to the knee, hand/finger, face, and dental. PMI injuries were most often an SR at the knee or a hand/finger fracture. This is in line with what has been reported for senior, U-18 and U-16 players in Denmark, except that they also reported high numbers of ankle injuries [14]. Handball is a physical sport with fast tempo, rapid changes of directions, and frequent contact and collisions between players [11]. Players do not use protective equipment, and mouth guards are rarely used [3]. Sidestep and landing manoeuvres are common injury mechanisms for knee injuries [16]. Since such manoeuvres are a necessary part of the sport, one needs to look at the methods of injury prevention, such as NMT. The implementation of such programs has had an injury preventive effect in different handball populations [15, 17]. The main injury mechanism for facial injuries is being hit by an arm or an elbow [18], and often caused by foul play, where only 60% of the events were sanctioned by the referee [12]. “Fair play”, attentive referees, and a mouth guard may prevent some of these injuries. In the present study, there was a decrease in knee and dental injuries after 2013. In 2014, there were rule-changes and clarifications of rules regarding offences and fair play that could impact the incidence of face and dental injuries. From 2013, there has been a gradual implementation of an NMT program to reduce injuries, especially knee injuries, nationwide in Sweden. This may have explained some of the reduction in knee injuries. In 2006, a rule change was introduced to reduce head injuries which may explain some of the reduction in dental injuries [2].

### Ice hockey

Ice hockey is a fast game on ice surrounded by rigid plastic and Plexiglas boards, where a player can reach speeds up to 48 km per hours and the puck over 160 km per hour. Due to high-speed collisions, the injury risk is proposed to be high [29]. The present study revealed that male ice hockey players had the highest incidence of injury in head/ neck body parts, and in most of the upper limb parts, compared to athletes in the other sports. PMI 1 + injuries occurred most often at the shoulder in males, and at the knee in females. The most common shoulder injury mechanism has been recognized to be body contact or body checking, which mainly affects the upper body and head/neck [1, 29]. Men are allowed to body-check the opponent, but not woman who are also required to wear full-face protection regardless of age. For men, the face protection rule terminates after the age of 18, when only a semi-covering protection, covering the eyes and nose, along with a gum-shield, is mandatory. It thus seems reasonable that males have a higher incidence of dental, facial, and shoulder injuries compared to female players, and other team players in the present study. It might also explain the increase in proportion of dental injuries in male hockey players after the age of 18 years. To reduce the frequency of head and upper limb impacts, player contact rules should be reconsidered, along with reinforcement of “fair play”. Helmet design could be revaluated to protect against high-magnitude head impacts caused by contact with the ice and boards [1, 13]. The use of flexible boards is shown to reduce concussions [29]. Female players, in agreement with a previous study, had a higher incidence of knee injuries and concussions [13]. They even had the highest incidence of concussion in all players in the present study. How these injuries should be prevented needs further investigations [13].

Different sports have different risk factors for injury. Team sports, like in this study, have opponents’ behaviour as one common risk factor. Contact with another player is one risk factor and also the manoeuvre to avoid contact is a risk factor, for example for a knee injury. In floorball, handball, and football, deliberately body contact is more or less prohibited and the avoidance of contact contributes to the high frequency of non-contact knee injuries in these sports [9, 10, 18]. Ice hockey players are allowed to have body contact which is the major injury mechanism during ice hockey matches [1, 24] involving high forces to the shoulder and accelerations to the head and neck. Handball players handle the ball with the hand which increases the risk for PMI injuries in the hand and fingers.

Advantages of using an insurance database in the present study are that it covered all licensed players at all the ages and levels of sports nationwide. Injury definition was the same, and the same systematic routines were used in all claims regardless of sport [30]. However, only those injuries reported to the insurance company were included in the study. The previous studies have shown that not all injuries are reported to insurance companies [7]. The health care system in Sweden is subsidized possibly reducing the need to report an injury claim. Dental health care, however, is less subsidized. This may have influenced the high incidence of a severe injuries and dental injuries. There was a decrease in dental injuries in all ball sports during the study period, especially after 2013. The cause for this is unknown, but severe dental injuries often take several years to treat and if the player reports the injury after the treatment is finished, then the injury will be reported late to the insurance company. Another consideration is the option to report an injury to the insurance company within 3 years (in rare cases up to 10 years) after it occurred. Injury data in the present study were exported from the insurance database at the end of 2016, 1 year after the end of the present study (year 2015). Approximately 80–90% of the injuries claims are reported to the insurance company within 1 year after occurrence, and thus, there is a risk that the injury incidences are somewhat underestimated for later years in the studied period. Moreover, the accuracy of the reported number of licensed players for each sport is dependent on the accuracy of the information from the different SFs, since the insurance company only records the injuries, not all licensed players within the SFs.

To the best of our knowledge, this is the first study to evaluate acute injury incidence over a 10-year period that includes all licensed players, both adolescent, amateur, and professional, in a whole nation. This makes this study unique and the results can guide coaches and medical staff as well as municipalities in their efforts to prevent acute sports injuries, at the local and national level.

## Conclusion

To achieve the greatest impact in reducing the adverse effects of acute sport injuries among licensed floorball, football, handball, and ice hockey players nationwide in Sweden, this study shows that preventive measures should focus on knee injuries in all the investigated sports. The severe head/face and upper limb injuries also need attention. There are suggested preventive interventions such as NMT training programs, safety equipment, and rule enforcements that have been shown to reduce the incidence of these injuries, but they need to be implemented, used, and evaluated nationwide.
